# Tong Xie Yao Fang: A Classic Chinese Medicine Prescription with Potential for the Treatment of Ulcerative Colitis

**DOI:** 10.1155/2021/5548764

**Published:** 2021-06-09

**Authors:** Kai Chen, Yu Lou, Ying Zhu

**Affiliations:** ^1^Hunan University of Chinese Medicine, Changsha, Hunan 410208, China; ^2^Department of Gastroenterology, The First Affiliated Hospital of Hunan University of Chinese Medicine, Changsha, Hunan 410007, China

## Abstract

The prescription of Tong Xie Yao Fang (TXYF) was derived from the Yuan dynasty “Dan Brook Heart Law,” which was a representative formula for treating liver-spleen disharmony, diarrhea, and abdominal pain. The prescription is composed of four herbs for soothing the liver and strengthening the spleen. TXYF is reportedly capable of eliminating discomfort in ulcerative colitis (UC). This classic formula has been widely used for regulating gastrointestinal motor dysfunction and repairing colon mucosa. This review aims to provide current information on the pharmacology and clinical research of TXYF in the treatment of UC, and to critically appraise that information, in order to guide the future clinical use and experimental study of TXYF in the treatment of UC. We searched online databases including PubMed, CNKI, and Google Scholar for research published between 2010 and 2020 on TXYF and its efficacy in the treatment of UC. The findings indicated that TXYF has anti-inflammatory and immunomodulatory effects, regulates cell signal transduction, brain-gut axis, and intestinal flora in UC, and may promote targeting of bone mesenchymal stem cells (BMSCs) to the colonic mucosa and accelerate healing of the colonic mucosal barrier. In addition, the results of clinical studies showed that TXYF has good efficacy and few adverse reactions in the treatment of UC. Although it has achieved some success, the research is limited by deficiencies; there is a lack of unified standards for the construction of UC animal models and for administration regimen. In addition, the dosage of TXYF is not consistent and lacks pharmacological verification, and clinical trial data are not detailed or sufficiently rigorous. Therefore, a more rigorous, comprehensive, and in-depth study of TXYF in the treatment of UC is needed.

## 1. Introduction

Inflammatory bowel disease (IBD) is a chronic inflammatory disease for which etiology is unknown [1], but may be related to genetic, immune, or environmental factors [2]. There are differences in microbial composition and epigenetic characteristics between inflamed and uninflamed colon fragments in IBD [3]. IBD is characterized by diarrhea, abdominal pain, or discomfort and blood in stool. Since 1990, the incidence of IBD has stabilized in the Western world, but newly industrialized countries are facing rising incidence [4]. IBD includes two forms, namely, ulcerative colitis (UC) and Crohn's disease (CD). UC mainly affects the colon and rectum [5] and the peak age of its onset is between 30 and 40 years [6]. Approximately 20% of patients with UC require hospitalization during the course of their disease [7], and up to 10% need to eventually undergo a colectomy [8]. Medicine is selected for patients with UC according to disease severity and may include amino salicylate, corticosteroids and immunosuppressants, anti-integrin agents, antitumor necrosis factor agents, or Janus-kinase inhibitors [9–11]. There are currently many new drugs under research and development, such as interleukin 23 antagonists. These novel therapies have shown promise for inducing and maintaining clinical benefits and have an excellent safety profile [12]. One systematic review found that infliximab is the most commonly used medicine in patients undergoing biological examination for the first time, while ustekinumab and tofacitinib are more commonly used in patients who have previously used antitumor inhibitors [13]. Antidepressants can improve functional symptoms and disease activity [14]. The treatment of UC is limited, and there are many side effects. Therefore, there is an urgent need for drugs that can effectively improve patients' symptoms and survival [15]. Traditional Chinese medicine (TCM) has been used to treat common, frequently occurring, and refractory diseases for thousands of years [16]; however, due to the lack of high-level evidence, its validity is often questioned [17]. TCM has multiple components and targets, and some Chinese medicines and their extracts have been found to improve symptoms and healing in UC by regulating the intestinal flora and improving the intestinal functional barrier [18].

TXYF is one of the most famous Chinese medicine prescriptions. It consists of four herbs (Table 1) and was first recorded in a book named “Dan Brook Heart Law” written by the students of Zhu Danxi in Yuan dynasty. According to the theory of Chinese medicine [19], it can be used to treat many diseases of liver–spleen discord, such as the liver-qi stagnation and spleen deficiency syndrome of irritable bowel syndrome (IBS) [20]. It has been used to treat abdominal pain and diarrhea in UC for several hundreds of years. TXYF is composed of Rhizoma Atractylodis Macrocephalae, Paeoniae Radix Alba, Citri Reticulatae Pericarpium, and Saposhnikoviae Radix. Rhizoma Atractylodis Macrocephalae and Citri Reticulatae Pericarpium strengthen the spleen to eliminate dampness while Paeoniae Radix Alba and Saposhnikoviae Radix soothe the liver to regulate qi [21]. TXYF improves colonic mucosal pathological tissue score and disease activity in UC; it regulates the migration, proliferation, and apoptosis of epithelial cells and contributes to mucosal healing in UC [22]. Increasingly, studies have shown the effectiveness of TXYF in treating ulcerative colitis [23], but the underlying treatment mechanism remains unknown. This review comprehensively explains the potential mechanism of TXYF in the treatment of ulcerative colitis and provides guidance for future research on classic prescriptions.

## 2. Pathophysiological Studies

**Figure 1:**
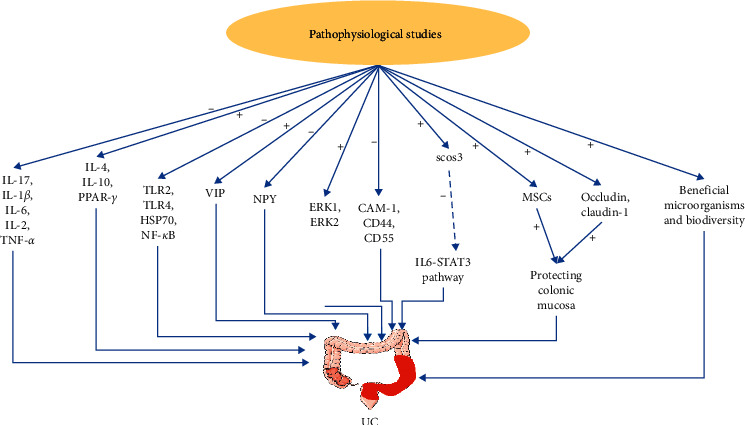
Pharmacological mechanism of Tong Xie Yao Fang in the treatment of ulcerative colitis. Tong Xie Yao Fang can alleviate colitis by activating or inhibiting pathways. +: activate; −: inhibit. IL-17: interleukin-17; IL-1*β*: interleukin-1*β*; IL-6: interleukin-6; IL-2: interleukin-2; TNF-*α*: tumor necrosis factor alpha; IL-4: interleukin-4; IL-10: interleukin-10; PPAR-*γ*: peroxisome proliferators-activated receptors-*γ*; TLR: toll-like receptor; HSP70: heat shock protein 70; NF-*κ*B: nuclear transcription factor-*κ*B; VIP: vasoactive intestinal peptide; NPY: neuropeptide Y; ERK: extracellular signal-regulated kinase; CAM: cell adhesion molecule; SOCS: suppressor of cytokine signaling; STAT: signal transducer and activator of transcription; and MSCs: mesenchymal stem cells.

Figure 1 illustrates the mechanism of TXYF related to immune balance and colon mucosa healing in the treatment of UC.

### 2.1. Anti-Inflammatory and Immunomodulatory Effects

#### 2.1.1. Inflammatory Factors

Inflammatory factors may be produced by mucosal immune cells and have a crucial role in the pathogenesis of IBD. One type of activated macrophages may aggravate IBD by producing a wide range of proinflammatory cytokines, such as tumor necrosis factor-*α* (TNF-*α*), interleukin-1*β* (IL-1*β*), and interleukin-6 (IL-6) [24]. The balance between pro- and anti-inflammatory cytokines is very important for the control of UC [25]. Anti-TNF is effective in inducing mucosal healing in UC, reflecting the important role of TNF in the pathogenesis of UC [26]. Numerous pieces of evidence indicate that the nuclear transcription factor-*κ*B (NF-*κ*B) pathway plays an essential role in pathogenic development of ulcerative colitis [27], with raised NF‐*κ*B levels in mucosal macrophages causing the production of proinflammatory cytokines, thus directly resulting in mucosal tissue damage [28]. In experiments using rats, expression of NF-*κ*B p65 protein and gene are reduced with TXYF treatment, indicating that it may have the effect of preventing overactivation of the NF-*κ*B signaling pathway [29]. TXYF inhibits the expression of inflammatory promoters as well as increasing the levels of inflammatory inhibitors, thereby regulating both pro- and anti-inflammatory factors. In a clinical trial including 62 UC patients, those in the control group were treated with sulfasalazine, and the treatment group was additionally treated with TXYF. After 1 month of treatment, the levels of interleukin-4 (IL-4) and interleukin-10 (IL-10) were significantly increased in both groups, while the levels of interleukin-17 (IL-17) and interferon-*γ* (IFN-*γ*) were decreased, the decrease trend was statistically in the treatment group compared with the control group [30]. The mechanism of TXYF in treatment of UC may involve inhibition of the expression of proinflammatory factors interleukin-2 (IL-2) and IL-6 in serum and increased expression of anti-inflammatory factor IL-10 [31, 32]. It may also downregulate the expression of IL-6 in hypothalamus tissues in rats as confirmed by reverse transcription-polymerase chain reaction (RT-PCR) and enzyme linked immunosorbent assay (ELISA) [33]. TXYF may have an overall regulatory effect by soothing the liver and strengthening the spleen, thereby inhibiting the expression of IL-1*β* and TNF-*α*, repairing colon tissue, and eliminating inflammation and ulcer [34].

#### 2.1.2. Toll-Like Receptor

Toll-like receptors (TLRs) play an important role in the innate immune system and participate in the inflammatory process of protein. Activation of TLR signaling pathways results in the induction of many genes that play a role in host defense, including inflammatory cytokines, chemokines, and antigen-presenting molecules. According to a recent perspective, TLR mutations and disorders are the main factors in IBD susceptibility and vulnerability, so their regulation may be a new and promising approach for the treatment of UC [35]. One of the therapeutic mechanisms of TXYF on UC in rats may be related to the inhibition of TLR4 in the upstream NF-*κ*B signaling pathway and the downstream expression of TNF-*α* and IL-1*β*. In rats with UC induced by the 2,4,6-trinitrobenzene sulfonic acid (TNBS)/ethanol enema method, TLR-4 in colonic mucosa, TNF-*α* and IL-*β* levels in serum were significantly higher than controls and were all decreased after four weeks of TXYF treatment [36]. In another experiment, 60 rats were divided into six groups, blank control group and model group treated over a 10-day period with TXYF at low-, medium-, or high-dose mesalazine or with no treatment. Heat shock protein (HSP70), TLR2, and TLR4 gene expressions were significantly lower in all TXYF treatment groups than in a model control group with no treatment. Treatment effect was highest in the medium-dose TXYF group, with a better effect than mesalazine, indicating that TXYF may inhibit the expression of HSP70 and TLRS genes in colonic mucosal tissue to treat ulcerative colitis [37].

#### 2.1.3. PPAR-*γ*

Peroxisome proliferators-activated receptors-*γ* (PPAR-*γ*) belong to the nuclear receptor superfamily of ligand-induced transcription factors and play an important role in the regulation of adipocyte differentiation, lipid metabolism, and insulin resistance. PPAR-*γ* relieves colonic inflammation by regulating the release of various proinflammatory factors. PPAR is mainly produced by colonic epithelial cells, and the activation of PPAR-*γ* has been shown to inhibit colonic inflammation and reduce disease severity in various experimental models of UC [38]. In an animal experiment induced by TNBS/ethanol enema, experimental animals were divided into 6 groups; PPAR-*γ* gene and protein expression levels were detected using RT-PCR and immunohistochemical methods. The relative expression levels of PPAR-*γ* gene and protein of colonic tissues of rats in the model group were significantly lower than those of the blank group. The relative expression of PPAR-*γ* gene and protein were increased after TXFY high and moderate doses. These results indicated that the relative expression levels of PPAR-*γ* gene and protein in colon tissue of rats were promoted by TXYF [39]. Another rat experiment showed that TXYF was effective in the treatment of experimental UC, and its mechanism may involve raising the antioxidant capacity of colon tissue, regulating the immune dysfunction of colon tissue, and increasing the expression of PPAR-*γ* in the colon [40].

### 2.2. Brain-Gut Axis

Patients with IBD have high rates of obsessive-compulsive disorder, panic disorder, depression, and anxiety [41]. Visceral hypersensitivity is the most widely known cause of abdominal pain, an important symptom in UC patients. The 5-hydroxytryptophan (5-HT) signaling pathway is important for both sensory signal transduction in gastrointestinal motility and the development of visceral hypersensitivity [42]. In one study, 60 male Sprague Dawley rats were randomly divided into blank control group, model group, TXYF low-, medium-, and high-dose group, and mesalazine group. After modeling and 4 weeks of drug intervention, 5-HT in the serum was measured using the ELISA method, and in the colon using PCR. The expression of 5-HT and 5-HT2 receptor increased significantly in the model group and reduced significantly after the TXYF intervention [43]. The role of 5-HT transporter (SERT) is to facilitate reuptake of 5-HT at the effector site, and the decrease of SERT expression leads to a decrease in 5-HT inactivation. TXYF downregulates liver 5-HT2R protein expression level through upregulation of colonic SERT protein, thereby regulating immune inflammation and promoting mucosal healing [44]. Neuropeptide Y (NPY) and vasoactive intestinal peptide (VIP) are two forms of neurotransmitter that play an important role in the pathogenesis of abnormalities in the brain-gut axis in irritable bowel syndrome (IBS). NPY is produced by T lymphocytes, macrophages, monocytes, and dendritic cells during inflammation, and it modulates the immune cell activities via paracrine or autocrine signaling [45]. VIP affects the secretion of cytokine and the synthesis of immune protein, plays an important role in stabilizing IL-10 mRNA in regulatory B cells (Bregs), and efficiently inhibits experimental colitis in mice [46]. In a UC rat experiment induced by TNBS/ethanol enema, after successful modeling, the level of VIP in serum was lower and the NPY level was higher in the model group than the blank group. After treatment, the level of VIP in TXYF group was higher and NPY lower than that in the model group. These findings indicate that upregulation of VIP concentration as well as downregulation of NPY concentration may be part of the mechanism in the treatment of UC [47].

### 2.3. Cell Signal Transduction

#### 2.3.1. ERK Signaling Pathway

Extracellular signal-regulated kinase (ERK) is a member of the mitogen-activated protein kinase (MAPK) family which is involved in the transmission of extracellular signals to intracellular proteins. The ERK/MAPK cascade is one of the four unique cascades of MAPK, which play an important role in the regulation of several fundamental processes, such as proliferation, differentiation, and cell responses to different external stresses [48]. The signaling pathways regulate various cellular processes, and abnormal regulation of ERK may be closely related to chronic inflammation and cancer. It is worth noting that ERK is significantly activated in UC, suggesting that the ERK signaling pathway may be correlated with the development of UC [49]. One study has shown that ERK attenuation reduces inflammation [50], while another showed that it inhibits signal transduction and activation of transcription 3 (STAT3) and Th17-cell differentiation [51], demonstrating involvement in the regulation of immune cell differentiation and contribution to the pathogenesis of colitis [52]. In a study on rats, after TNBS/enema, the animals were observed for morphological injury and damage, and RT-PCR was used to detect ERK1 and ERK2 gene expressions. Expression levels in colonic tissues were significantly higher in the model group than in the blank group and were significantly increased in rats treated with high or moderate doses of TXYF compared with the model group. This indicates that the therapeutic effects of TXYF are probably related to activation of the ERK signal transduction pathway [53].

#### 2.3.2. CAM

Cell adhesion molecules (CAMs) are part of the immunoglobulin superfamily and are required for the combination between leukocytes with tissue components as well as leukocytes with inflamed areas [54, 55]. The four main types of CAM are ICAM-2, ICM-1, MAdCAM-1, and VCAM-1, of which intercellular adhesion molecule 1 (ICAM-1) is the most important since its upregulation in endothelial cell wounds increases the accumulation of epithelial-associated neutrophil and leads to injury of the colon mucosa in UC [56, 57]. Vedolizumab is a specific monoclonal antibody which can selectively block the combination of mucosal addressin cellular adhesion molecule 1 (MAdCAM-1) and integrin *α*4*β*7 and is effective in the induction and maintenance of remission in UC and CD [58]. Similarly, ICAM-1 antisense oligonucleotide has shown efficacy in relieving symptoms as well as improving biochemical indicators in experimental models [59]. In an animal experiment, sixty specific pathogen-free (SPF) Wistar rats were randomly divided into a blank group, model group, TXYF low-, moderate-, high-dose groups, and sulfasalazine (SASP) group and treated for 21 days. Apart from the blank group, the UC models were established by TNBS/ethanol enema, after which the TXYF low, moderate, and high doses were administered at 11, 22 and 44 g·kg^−1^, respectively, by gavage, while SASP was administered at 0.3 g·kg^−1^ by gavage, and the blank and model groups were treated with the same volumes of physiological saline. The expression amounts of ICAM-1 gene and protein in colonic tissues of rats in the model group were significantly higher than those in the blank group, while expression was significantly lower in the TXYF high- and moderate-dose groups. These results indicate that TXYF downregulates the expression of ICAM-1 mRNA and protein in colon tissue, thus inhibiting the infiltration of inflammatory cells, as well as repairing the damaged colon tissue [60]. The CD44 family is an important member of CAM, being a transmembrane glycoprotein which is overexpressed on the surface of activated macrophages in colon tissue [61]. It is involved in the pathogenesis of tumors and is thought to be one of the markers on the tumor surface [62]. Similarly, the expression of CD44 is increased in UC [63, 64]. Anti-inflammatory drug-loaded nanoparticles delivered through the CD44-mediated endocytosis pathway have shown effectiveness in the treatment of UC [65]. In a rat experiment, TXYF downregulates the expression of CD44 and CD54, inhibits local inflammatory mediators, decreases the release of chemotaxis, and thus reduces the inflammatory response of the intestinal mucosa [66].

#### 2.3.3. JAK/STAT3 Signaling Pathway

According to current research, signal transducer and activator of transcription (STAT) 3 is known to be related to colonic inflammation and is activated by different cytokines and growth factors [67]. Increased STAT3 phosphorylation at tyrosine residues is found in the murine dextran sulphate sodium- (DSS-) induced colitis model, as well as in the epithelial tissue and lamina propria cells of IBD patients [68]. Suppressor of cytokine signaling (SOCS) is a family of proteins regulating negative feedback to the Janus-kinase (JAK)/STAT signaling pathway. SOCS3 is an important member of this family, being expressed in intestinal epithelial cells and lamina propria in both mouse colitis models and patients with UC, thus playing an important role in the pathogenesis of colitis [69]. The IL6-IL6R-STAT3-SOCS3 signaling pathway is known to play important roles in regulating intestinal epithelial homeostasis, in pathogenesis of IBD, and in tumorigenesis of colorectal neoplasia [70]. In an animal experiment, rats were randomly allocated to control, model, TXYF high-, medium-, and low-dose groups and mesalazine group, with 15 rats in each group. The concentration of serum IL-6 and the expression level of colonic tissue STAT3 gene and protein in model group rats were significantly increased compared with the control group which demonstrates the success of the UC model. The expression level of serum IL-6, and colonic STAT3 gene and protein in both TXYF high-dose group and mesalazine group were significantly lower than those in the model group. This indicates that the mechanism of TXYF in the treatment of UC may involve inhibition of the IL-6/JAK/STAT3 signal transduction pathway [71]. In another animal experiment, TXYF downregulated the expression level of glycoprotein 130 and upregulated the level of SOCS3 in colonic tissue, thereby effectively reducing the colonic mucosa damage of UC rats with liver depression and spleen deficiency [72]. These findings indicate that TXYF is involved in the regulation of the IL6-STAT3-SOCS3 signaling pathway, thus contributing to healing in UC.

### 2.4. Regulation of Intestinal Microbiota

Gut microbes play an important role in the pathogenesis of UC, probably linked to the close relationship between mucosal damage and the inflammatory response [73]. Intestinal flora imbalance between beneficial and harmful bacteria increases the permeability of intestinal epithelium, leading to the occurrence of IBD [74]. The beneficial bacteria (e.g., lactobacillus and bifidobacterium) protect the body from pathogenic microorganisms [75], and when these bacteria are insufficient, the harmful bacteria may induce lesions leading to the destruction of mucosal barrier function and increased intestinal permeability [76]. In addition, the imbalance between host and intestinal flora may lead to an inflammatory response in UC [49, 77] (e.g., TNF-*α*, IL-6, and IL-1b). In a rat experiment, TXYF significantly reduced the similarity between samples as well as increasing the level of intestinal probiotics (e.g., o-Lactobacillales) compared with a model group, helping to repair intestinal damage and balance the local microenvironment in the intestine [78]. There are few studies about the effect of TXYF on intestinal microecology; thus, more in-depth and comprehensive research on gastrointestinal microflora and TXYF in UC is needed in the future.

### 2.5. MSCs

Mesenchymal stem cells (MSCs) have the potential to differentiate into cell types that can repair damaged mucosa, via inhibition of inflammatory factors and downregulation of inflammatory response [79]. A systematic review and meta-analysis confirmed the effectiveness and safety of MSCs in the treatment of UC [80], indicating that targeted MSCs hold promise for future application [81]. In a rat experiment induced by TNBS/ethanol enema, intragastric TXYF was administered once a day for 28 days. After administration, peripheral blood was separated and purified, and BMSCs with 4′,6-diamidino-2-phenylindole- (DAPI-) labeled BMSCs suspension were injected through the tail vein. On the 7th and 14th days, the distribution of BMSCs in the rat colonic mucosa was observed by laser confocal microscopy, and the colonic tissue morphology was observed by hematoxylin-eosin (HE) staining. The results showed that in rats treated with TXYF the pathological state of colon tissue was improved and the distribution of BMSCs in the colon mucosa was increased compared with a model group. Similarly, another experiment found that TXYF promotes the growth and proliferation of BMSCs from bone marrow and peripheral blood and ensures a sufficient number of BMSCs native to the colonic mucosa to play a role in repair, which may be a mechanism in the treatment of UC [82, 83].

### 2.6. Colon Mucosa Barrier

There is a close relationship between epithelial cells and tight junctions [84]. Tight junctions are crucial for intestinal barrier function; they can segregate gut microbiota and the host immune system to avoid inappropriate immune responses to gut microbes and can prevent pathogens and antigens from entering the body [85]. Tight integration of intestinal epithelial cells is essential in IBD, and impairment of the intestinal barrier and absorption dysfunction are associated with a series of clinical symptoms [86, 87]. Occludin and occludin 1 are the main protein components of tight junctions, their removal from tight junctions, or reduced expression leading to colonic barrier loss and breakdown [88]. In one experiment, forty patients with UC were randomly divided into control group (*n* = 20) and observation group. The control group was treated with mesalazine enteric-coated tablet orally, the observation group was additionally treated with TXYF, and the course of treatment for both groups was 12 weeks. Expression of occludin, claudin-1, and *β*-defensin in colonic mucosa was detected. The results showed that the expression levels of occludin and claudin-1 of colonic mucosa in the observation group were significantly higher than those in the control group, while the expression level of *β*-defensin in the observation group was significantly lower. These results indicate that TXYF increases the expression of protective factors of the intestinal mucosa barrier, thus promoting intestinal mucosa healing in patients with UC [89]. This result was validated in an experiment on rats [90].

## 3. Clinical Research

TXYF has the effect of soothing the liver and strengthening the spleen, which can effectively relieve abdominal pain, diarrhea, and other clinical symptoms in patients with UC. It can relieve clinical symptoms in UC patients and improve the pathological score under endoscopy when used as a monotherapy or combined with other classic formulae. It can also be used as a retention enema targeting local damaged colon mucosa directly, thus promoting the healing of colon inflammation and relieving the symptoms of patients with UC. The combination of Western medicine and TXYF can reduce the dosage of Western medicine and any related adverse reactions [91]. TXYF can also reduce anxiety and depression in patients with UC when used as an adjuvant therapy [92]. Representative clinical studies are shown in Table 2.

## 4. Chemical Constituents of TXYF

**Figure 2:**
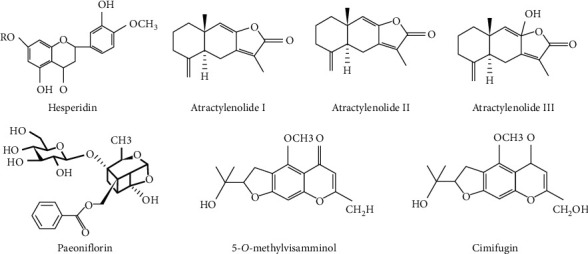
The chemical structure of the main active components of Tong Xie Yao Fang. Hesperidin is the main active component of Citri Reticulatae Pericarpium (chen pi). Atractylodes macrocephala Koidz (baizhu) consists of atractylenolide I, atractylenolide II, and atractylenolide III. The main active component of Paeoniae Radix Alba (Bai shao) is Paeoniflorin. Saposhnikovia divaricate (fangfeng) includes 5-*O*-methylvisamminol and cimifugin.

The combination of rhizomes, roots, and peel of various herbs includes different chemical structures. Citri Reticulatae Pericarpium (chen pi) contains multiple chemical components, including flavonoids, limonin, alkaloids, and volatile oils. Hesperidin is one constituent of dihydroflavone glycosides, which is used as an indicator of Citri Reticulatae Pericarpium [102, 103]. The major chemical constituents of *Atractylodes macrocephala* Koidz (baizhu) are volatile oils (Atractylon, Aromadendrene, and Elemene) and polysaccharides. Atractylenolide is the main ingredient in baizhu and consists largely of atractylenolide, atractylenolide II and atractylenolide III [104]. Paeoniae Radix Alba (bai shao) is derived from the dried root of Paeonia lactiflora Pall and mainly contains monoterpene glycosides, triterpenoids, flavonoids, tannins, and other chemical constituents. At present, 140 chemical constituents have been isolated from Radix Paeoniae Alba. A chemical component of monoterpene glycosides, namely, the total glucosides of paeony (TGP), is the main physiologically active substance in Radix Paeoniae Alba, paeoniflorin occupying the highest proportion at 3% [105]. The chemical composition of Saposhnikovia divaricate (fangfeng) is very complex, and its main active substances are ketone, coumarin, volatile oil, and other ingredients. Cimicifugin and 5-*O*-methylvisammiol are the main chemical constituents of ketone [106]. The chemical formula of the representative components of Tong Xie Yao Fang is shown in Figure 2. A reliable ultraperformance liquid chromatography-tandem mass spectrometry (UPLC-MS/MS) method has been developed and validated for sensitive and rapid determination of multiple analytes from TXYF decoction in three biological matrices. Using this method, 1 lactone, 2 monoterpene glucosides, 1 alkaloid, 5 flavonoids, and 2 chromones were detected and quantified in plasma, brain tissue, and urine after oral administration of TXYF decoction [107].

## 5. Summary and Outlook

UC is a refractory and recurring disease with complex etiology and involves multiple mechanisms [108]. The immunomodulator agents derived from herbal medicine have shown potential for application in UC, and this has been confirmed by mechanisms demonstrated in many clinical and animal studies [109]. Multitarget combination therapy is an approach with potential for broad application in the future. TXYF is a classic formula including four herbs containing hundreds of chemical ingredients and effectively attenuating disease activity through different mechanisms demonstrated experimentally and may provide options for multitargeted therapy.

The results of pharmacological studies have shown that TXYF has the effects of regulating the balance between anti- and proinflammatory factors, downregulating the expression of TLRs, and upregulating PPAR-*γ*. TXYF improved related indices in the brain-gut axis and promoted targeting of MSC to colonic mucosa and healing of the colonic mucosal barrier. TXYF regulates cell signal transduction through the activation of ERK and downregulation of cell adhesion molecule expression; the IL6-STAT3-SOCS3 signaling pathway may be another cellular pathway for TXYF treatment of UC. TXYF may increase the number of intestinal probiotics and regulate the diversity of intestinal flora. Clinical studies have shown that TXYF alleviates discomfort in patients with UC, reduces disease activity and pathological change in endoscopic colon tissue, and regulates the expression of some inflammatory cytokines as well.

Although some studies have shown that TXYF is effective in the treatment of UC, they have suffered from some limitations as follows: (1) A DSS-induced colitis model is most widely used in research on UC; however, most of the rats used in the research on TXYF treatment for UC were induced by TNBS/ethanol enema, and animal models are added with environmental interference factors which has personal subjectivity. (2) The dosage of therapeutic drugs for UC is not consistent across studies and is based on convention, without pharmacodynamic verification. (3) The design of clinical trials has not been sufficiently rigorous; although groups were randomized, the process of grouping, treatment, and follow-up was not explained in detail. Most of them are open trials, and more randomized double-blind trials should be designed. (4) Experiments to date have not thoroughly investigated the pharmacological mechanism of TXYF in UC, the findings in rats have not been verified in vitro or in humans, and more in vitro and human studies are needed to verify the results.

In conclusion, past studies have demonstrated the efficacy of TXYF in the treatment of ulcerative colitis. More in-depth, high-quality, and rigorous research on TXYF in the future may help to verify it as a supplement for the prevention or treatment of UC.

## Figures and Tables

**Table 1:** The formulation of Tong Xie Yao Fang (one dose).

Herbal (local name)	Medicinal part	Amount in application (g)
Atractylodes macrocephala Koidz (Bai zhu)	Rhizomes	12
Paeoniae Radix Alba (Bai shao)	Root	12
Citri Reticulatae Pericarpium (chen pi)	Peel	9
Saposhnikoviae Radix (fang feng)	Root	6

**Table 2:** Overview of clinical studies of TXYF.

Study drug	Number of UC patients	Experiment method	Observation of efficacy	Result
TXYF [93]	100	TXYF and bifidobacterium tablets were taken orally twice a day in treatment and control group for 4 weeks.	Clinical efficacy	The response in the treatment group is higher than control group (*p* < 0.05).

TXYF and SJZT [94]	60	TXYF in combination with SJZT was taken orally twice a day in treatment group and mesalazine four times a day in control group for 8 weeks.	Clinical and symptom effect	The effective clinical and efficacy of symptoms rate was higher in treatment group than control group (*p* < 0.05).

TXYF [91]	80	SAPA was taken orally three times a day in control group; amount was reduced after remission. Oral TXYF was taken twice a day in treatment group on the basis of control group drug halved. The course of treatment was 3 months.	Incidence of adverse reactions and level of C-reactive protein	The CRP level of treatment group was lower than the control group (*p* < 0.05), and there were fewer adverse events in treatment group (*p* < 0.05).

TXYF and THXWT [95]	80	Oral TXYF in combination with THXWT decoction twice a day in treatment group and SAPA three times a day in control group for 30 days; both groups were followed up for 1 year.	Clinical and electronic colonoscopy efficacy and recurrence rate	The clinical and colonoscopy efficacy rate in treatment group is higher than control group (*p* < 0.05). Recurrence rate under endoscopy in treatment group is lower than control group (*p* < 0.05).

TXYF [96]	68	TXYF twice per day in treatment group, mesalazine 3 times a day in control group for 8 weeks.	Clinical efficacy	The effective rate was higher in treatment group than control group (*p* < 0.05).

TXYF [97]	80	SAPA four times a day and prednisone 3 times a day in control group, oral TXYF in combination with SLBZS twice a day in treatment group. The total course of treatment was 4 weeks.	Clinical and electronic colonoscopy efficacy	The clinical and electronic colonoscopy efficacy is superior in treatment group than control group (*p* < 0.05).

TXYF [98]	82	Mesalazine four times a day in control group, TXYF is orally given to the treatment group twice a day on the basis of control group, the total course of treatment was 16 weeks.	Clinical efficacy rate and incidence of adverse reactions	The clinical efficacy rate is higher in treatment group than control (*p* < 0.05); there is no significant difference in the incidence of adverse reaction (*p* < 0.05).

TXYF [99]	56	Mesalazine anal suppository 3 times a day in control group. Oral TXYF twice a day in the treatment group on the basis of control group. The course of treatment was 60 days.	Clinical efficacy rate and interleukin level in serum	The effective rate is higher in treatment group than control group (*p* < 0.05), the level of IL-10 in treatment group is higher than control group, and the lever of IL-17 is lower (*p* < 0.05).

TXYF [100]	80	SAPA three times a day in control group, TXYF is orally given to the treatment group on the basis of control group, the total course of treatment was 8 weeks.	Clinical efficacy rate, DAI, ESR, and CRP	The clinical effective rate was higher in treatment group than control group (*p* < 0.05). The levels of DAI, ESR, and CRP improved significantly compared with the control group (*p* < 0.05).

TXYF [101]	90	SAPA 3 times a day and bifidobacterium triplex capsule 2 times a day in control group. TXYF is orally given to the treatment group on the basis of control group, the total course of treatment was 8 weeks.	Clinical efficacy rate and serological markers of inflammation	The effective rate was higher in treatment group than control group (*p* < 0.05); the levels of IL-1, IL-8, and TNF-*α* in the treatment group were significantly lower than control group (*p* < 0.05).

TXYF: Tong Xie Yao Fang; THXWT: Tao Hong Si Wu decoction; SLZBS: Shen Ling Bai Zhu powder; DAI: disease activity index; ESR: erythrocyte sedimentation rate; CRP: C-reactive protein; UC; ulcerative colitis; IL-1: interleukin-1; IL-8: interleukin-8; IL-10: interleukin-10; IL-17: interleukin-17; SASP: salicylazosulfapyridine; SJZT: Sijunzi decoction; and TNF-*α*: tumor necrosis factor-*α*.

## Data Availability

No data were used to support this study.

## Abbreviations

TXYF: Tong Xie Yao Fang
UC: Ulcerative colitis
BMSCs: Bone mesenchymal stem cells
IBD: Inflammatory bowel disease
CD: Crohn's disease
TCM: Traditional Chinese medicine
TNF-*α*: Tumor necrosis factor alpha
IL-1*β*: Interleukin-1*β*
IL-6: Interleukin-6
NF-*κ*B: Nuclear transcription factor-*κ*B
IL-4: Interleukin-4
IL-10: Interleukin-10
IL-17: Interleukin-17
IFN-*γ*: Interferon-*γ*
IL-2: Interleukin-2
RT-PCR: Reverse transcription-polymerase chain reaction
ELISA: Enzyme-linked immunosorbent assay
TLRs: Toll-like receptors
TNBS: 2,4,6-Trinitrobenzene sulfonic acid
HSP70: Heat shock protein 70
PPAR-*γ*: Peroxisome proliferators-activated receptors-*γ*
5-HT: 5-Hydroxytryptophan
SERT: 5-Hydroxytryptophan transporter
NPY: Neuropeptide Y
VIP: Vasoactive intestinal peptide
IBS: Irritable bowel syndrome
ERK: Extracellular signal-regulated kinase
MAPK: Mitogen-activated protein kinase
STAT3: Activator of transcription3
CAMs: Cell adhesion molecules
ICAM: Intercellular adhesion molecule
MAdCAM: Mucosal addressin cellular adhesion molecule
SPF: Specific pathogens free
SASP: Sulfasalazine
STAT: Signal transducer and activator of transcription
DSS: Dextran sulphate sodium
SOCS: Suppressor of cytokine signaling
MSCs: Mesenchymal stem cells
DAPI: 4′,6-Diamidino-2-phenylindole
HE: Hematoxylin-eosin
UPLC-MS/MS: Ultraperformance liquid chromatography-tandem mass spectrometry.
